# 325. Comparison of nasopharyngeal and oropharyngeal specimens for detecting Streptococcus pneumoniae colonization in children using molecular methods

**DOI:** 10.1093/ofid/ofaf695.114

**Published:** 2026-01-11

**Authors:** Liset Olarte, Naisha R Mekala, Lauren M Sommer, Linda Lamberth, David Hulten, Denver Niles, Sheldon L Kaplan, Kristina G Hulten

**Affiliations:** Baylor College of Medicine, Houston, TX; Rice University, Houston, Texas; Baylor College of Medicine, Houston, TX; Baylor College of Medicine, Houston, TX; Baylor College of Medicine, Houston, TX; Baylor College of Medicine, Houston, TX; Baylor College of Medicine, Houston, TX; Baylor College of Medicine, Houston, TX

## Abstract

**Background:**

Nasopharyngeal (NP) samples are considered the gold standard for detecting *Streptococcus pneumoniae* (SPN) colonization in children, while oropharyngeal (OP) sampling is more common in adults. The 2013 WHO guidelines suggest that in children OP cultures provide minimal additional yield to NP cultures. However, NP samples alone may underestimate colonization and serotype prevalence. While molecular methods are increasingly used, data on their reliability for OP samples in children is limited. We characterized the NP and OP pneumococcal colonization rate and serotype distribution in children.

**Figure:**
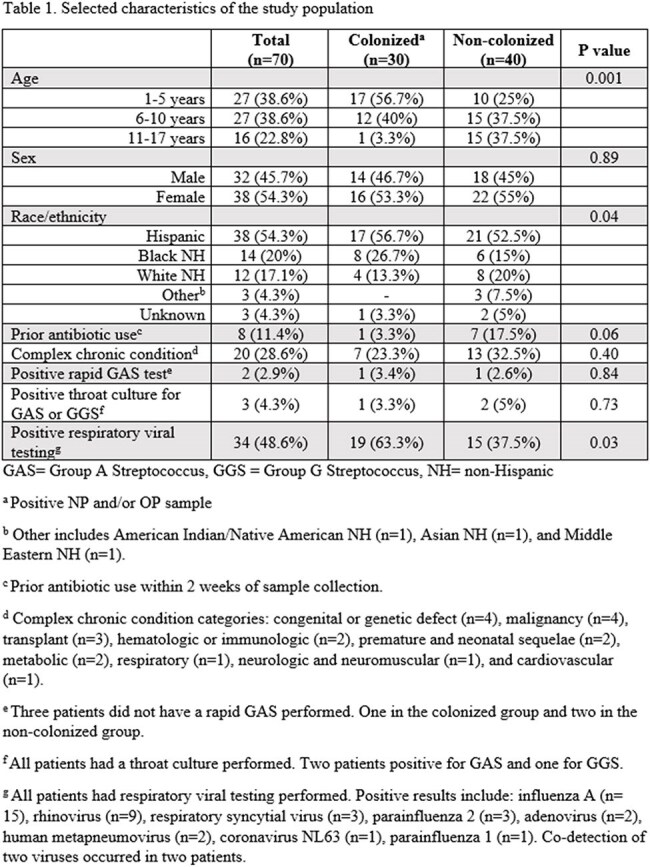

**Figure:**
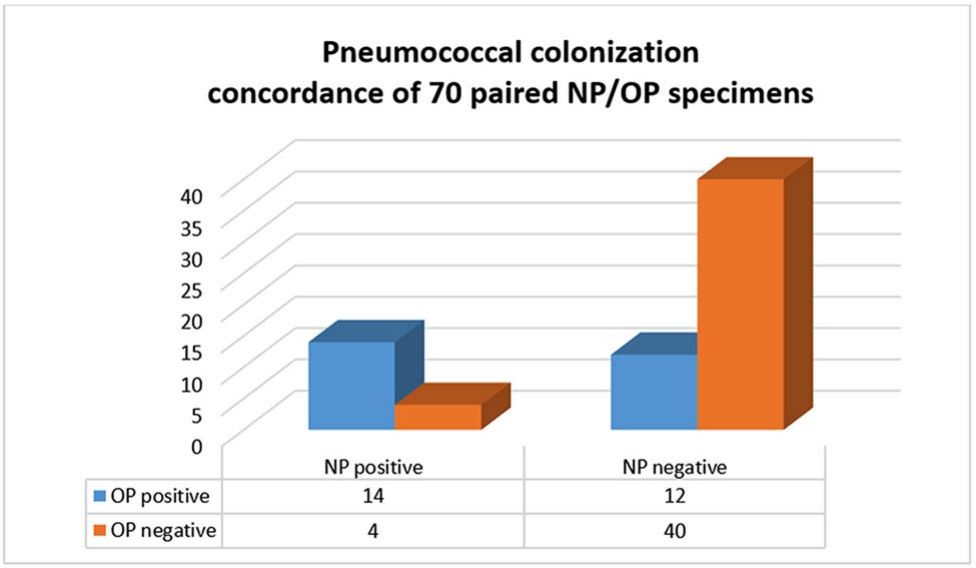

**Methods:**

This retrospective study used residual paired NP/OP samples, obtained as part of clinical care, from children < 18 years at Texas Children’s Hospital in December 2024. Pneumococcal colonization was assessed using a multiplex qPCR assay targeting *lytA*, *piaB*, and *SP2020* genes. Samples were run in duplicates, and positivity was defined as a Ct < 35 for ≥ 2 target genes. Samples with a Ct ≤ 32 (indicating a DNA concentration sufficient for the downstream PCR assays) underwent molecular serotyping using 8 sequential multiplex PCR assays. Demographic and clinical data were extracted from medical records.

**Figure:**
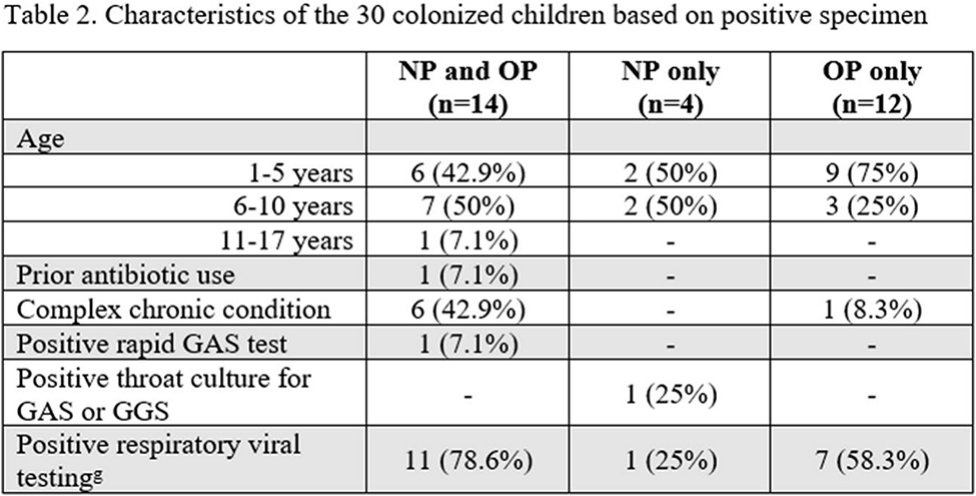

**Figure:**
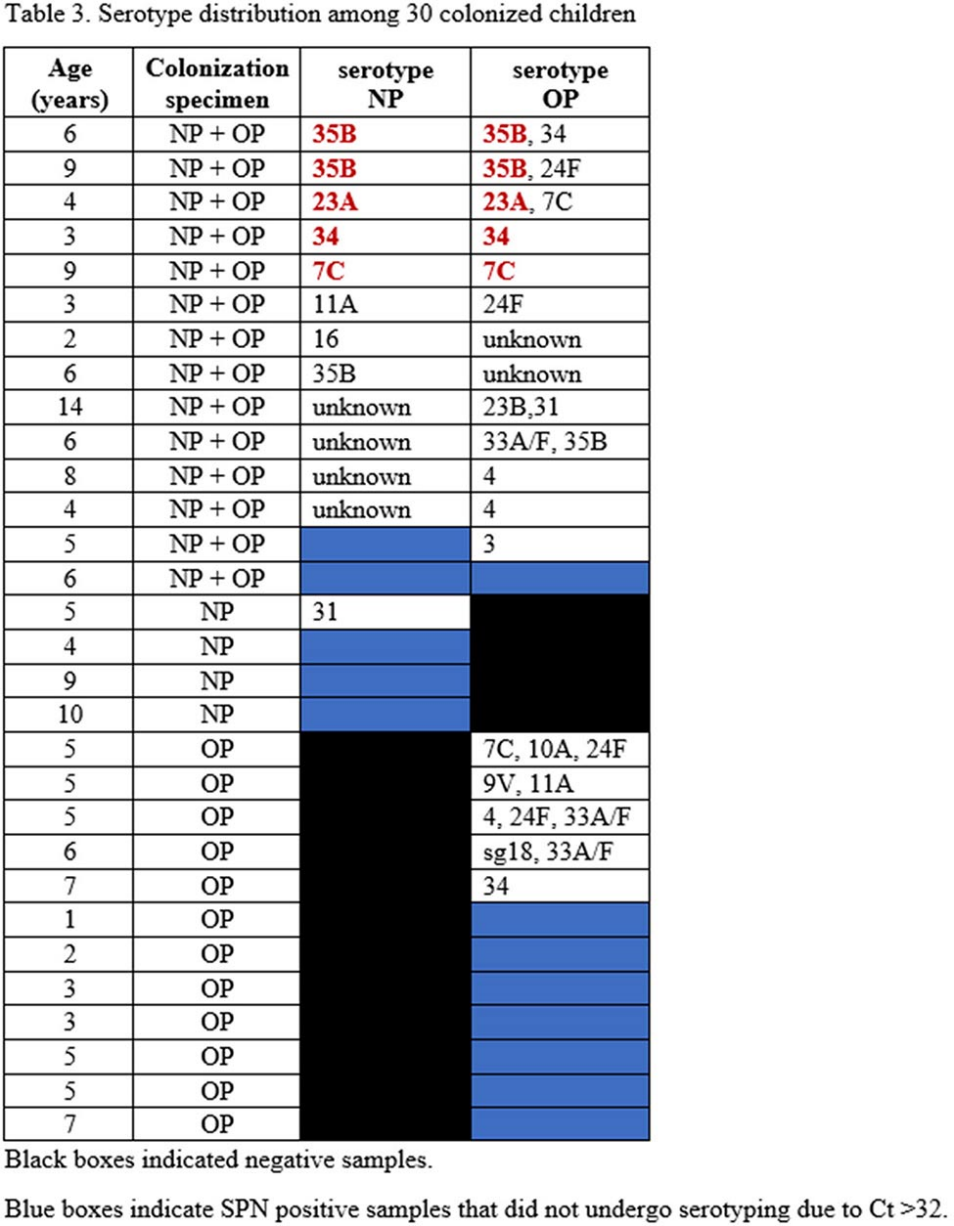

**Results:**

Seventy children with paired NP/OP samples were identified (Table 1). A total of 44 samples (18 NP and 26 OP) from 30 children were SPN positive, a 42.9% colonization rate. Positive concordance between NP and OP samples was observed in 20% of children, while 57.1% showed negative concordance (Figure). Colonized children were younger and more likely to test positive for respiratory viruses (Table 1). The characteristics of colonized children based on positive specimens are shown in Table 2. Fifteen different serotypes were identified, the most common from sites combined were 35B (n=6), 24F (n=4), 34 (n=4), and 7C (n=4). Co-detection of serotypes as well as serotypes 3, 4, 9V, 10A, sg18, 23B, 24F and 33A/F were noted in OP samples only. (Table 3).

**Conclusion:**

The addition of OP to NP samples identified 17.1% more SPN colonization and a broader serotype distribution. However, OP colonization results should be interpreted cautiously, as some serotypes (e.g. 4, 9V) are rare in children, and may reflect cross-reaction with non-pneumococcal streptococci carrying pneumococcal capsular genes.

**Disclosures:**

Liset Olarte, MD, MSc, GSK: Grant/Research Support|Merck, Sharpe & Dohme: Grant/Research Support|Sanofi: Grant/Research Support|UpToDate - WoltersKluwer: Royalties

